# Comparative efficacy, safety, and acceptability of single-agent poly (ADP-ribose) polymerase (PARP) inhibitors in *BRCA*-mutated *HER2*-negative metastatic or advanced breast cancer: a network meta-analysis

**DOI:** 10.18632/aging.202152

**Published:** 2020-11-30

**Authors:** Ju Wang, Ye Zhang, Long Yuan, Lin Ren, Yi Zhang, Xiaowei Qi

**Affiliations:** 1 Chongqing Municipal Center for Disease Control and Prevention, Chongqing, P.R. China; 2 Department of Breast and Thyroid Surgery, Southwest Hospital, Third Military Medical University (Army Medical University), Chongqing, P.R. China

**Keywords:** ADP-ribose, PARPi, breast cancer, efficacy, network meta-analysis

## Abstract

Background: Breast cancer is the most commonly diagnosed cancer and is the leading cause of cancer death in women worldwide. Both talazoparib and olaparib are approved by the US Food and Drug Administration for treating *BRCA* (breast cancer 1, early onset)-mutated *HER2* (human epidermal growth factor receptor 2)-negative metastatic or advanced breast cancer. However, the optimal choice of first-line treatment has not been determined.

Objective: To compare the efficacy, safety, and acceptability of single-agent poly (ADP-ribose) polymerase (PARP) inhibitors for patients with *BRCA*-mutated *HER2*-negative metastatic or advanced breast cancer.

Results: We included two trials comprising 733 participants. Compared with talazoparib, olaparib was not associated with improved PFS (*HR* = 1.08, 95% *CrI* = 0.34–3.45) or OS (*HR* = 1.18, 95% *CrI* = 0.61–2.31). Compared with talazoparib, olaparib was associated with non-significantly improved ORR (*OR* = 0.83, 95% *CrI* = 0.05–12.64). Regarding safety, olaparib had reduced risk for both grade 3–4 anemia (*OR* = 0.34, 95% *CrI* = 0.003–34.94) and any-grade anemia (*OR* = 0.37, 95% *CrI* = 0.02–6.81) compared with talazoparib. Olaparib also showed a low risk for grade 3–4 neutropenia (*OR* = 0.57, 95% *CrI* = 0.06–5.75) compared with talazoparib. Both talazoparib and olaparib were not associated with high risk of treatment discontinuation (*OR* = 0.95, 95% *CrI* = 0.21–4.47). Regarding time to QoL deterioration, olaparib was associated with short time to clinically meaningful QoL deterioration (*HR* = 1.16, 95% *CrI* = 0.19–7.17) compared to talazoparib.

Conclusion: Both talazoparib and olaparib have similar efficacy, safety, and acceptability in patients with *BRCA*-mutated *HER2*-negative metastatic or advanced breast cancer. Well-designed head-to-head randomized controlled trials with large samples are suggested to determine the optimal treatment choice.

Methods: We performed a systematic review and network meta-analysis. We performed a systematic search of Web of Science, Embase, PubMed, Medline, ClinicalTrials.gov, the Cochrane Central Register of Controlled Trials, and the World Health Organization International Clinical Trials Registry Platform, and international registers for published and unpublished double-blind randomized controlled trials from database inception to July 20, 2019. The pooled estimates of hazard ratios (HR) with 95% credible intervals (CrIs) were calculated for PFS, OS, and the time to deterioration of quality of life (QoL). The pooled estimates of odds ratio (*OR*) with 95% CrIs were calculated for ORR, AEs, and treatment discontinuation. This study is registered with PROSPERO (CRD42019138939).

## INTRODUCTION

Breast cancer is the most commonly diagnosed cancer, and is the leading cause of cancer death worldwide in women [1, 2]. There were approximately 2.1 million newly diagnosed female breast cancer cases in 2018, accounting for almost one-quarter of cancer cases among women worldwide [1].

Many factors increase the incidence of breast cancer, and its etiology is diverse. The cause of breast cancer is probably related to polygenic genetic factors [3, 4] and a complicated association of environmental factors [5]. Germline *BRCA1*/*2* (breast cancer 1/2, early onset) mutations have long been considered an important risk factor for breast cancer [6]. The inheritance of mutated *BRCA1* or *BRCA2* alleles results in a lifetime risk of breast cancer as high as 80% [7]. Furthermore, more than three-quarters of germline *BRCA* (g*BRCA*) mutant breast cancers have a molecular typing of triple-negative breast cancer (TNBC) [8]. The *BRCA1* and *BRCA2* genes are directly related to hereditary breast cancer [9], and are more frequently present in patients with a family history of breast cancer. Besides, *BRCA1*/*2* are tumor suppressor genes [10] that inhibit malignant tumorigenesis and encode proteins involved in repairing DNA double-strand breaks via the homologous recombination repair pathway [11].

Members of the poly (ADP-ribose) polymerase (PARP) family of nuclear enzymes play key roles in recognizing and repairing DNA single-strand breaks [10]. Under these mechanisms, preclinical studies have found that *BRCA1*/*2*-deficient cancer cells are sensitive to PARP inhibition [2, 12, 13]. Therefore, PARP inhibitors (PARPi) present new g*BRCA* mutation-targeted approaches in advanced breast cancer [14, 15]. Currently, two PARPi agents (olaparib and talazoparib) have received US Food and Drug Administration (FDA) approval for use in patients with g*BRCA*-mutated *HER2* (erb-b2 receptor tyrosine kinase 2)-negative advanced breast cancer previously treated with chemotherapy and/or endocrine therapy, respectively [14].

Talazoparib is considered a reasonable first-line option for advanced breast cancer, as is olaparib. Therefore, the optimal choice of first-line treatment has not been determined. However, to our best knowledge, there have been no head-to-head trials comparing single-agent PARPi in *BRCA*-mutated *HER2*-negative advanced breast cancer [14], and only one traditional meta-analysis has compared all single-agent PARPi with treatment of physician’s choice (TPC) [15]. The studies do not allow simultaneous comparison of all single-agent PARPi, constraining comparative assessment of the longer-term benefits and risks associated with available promising therapy. Therefore, we performed a network meta-analysis (NMA) to compare single-agent PARPi for advanced breast cancer.

## RESULTS

### Search results and study characteristics

**Figure 1:**
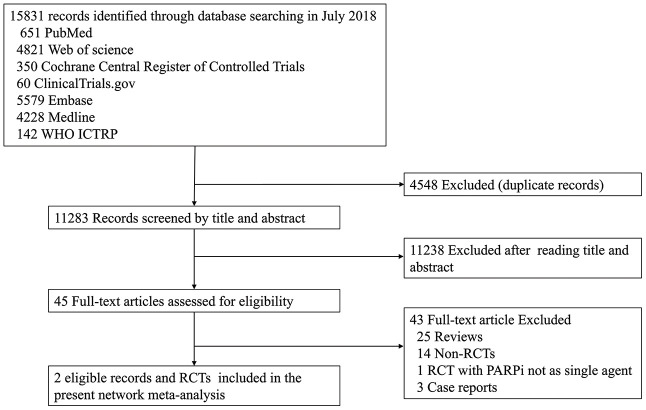
**The PRISMA flow chart summarizing the identification process of eligible studies.** WHO ICTRP, World Health Organization International Clinical Trials Registry Platform; PARPi, PARP inhibitor; RCT, randomized controlled trial.

Overall, the database search identified 15,831 studies; 4,548 studies were excluded due to duplicate records and 11,238 studies were excluded based on the selection criteria after reading the title and abstract. Subsequently, 45 potentially relevant full-text articles were reviewed. After applying all the eligibility criteria, 43 studies were excluded because 25 were reviews, 14 were non-RCTs, three were case-reports, and one were not single-agent PARPi studies. Therefore, two RCTs, the EMBRACA and OlympiAD, were included in our NMA (Figure 1).

**Figure 2:**
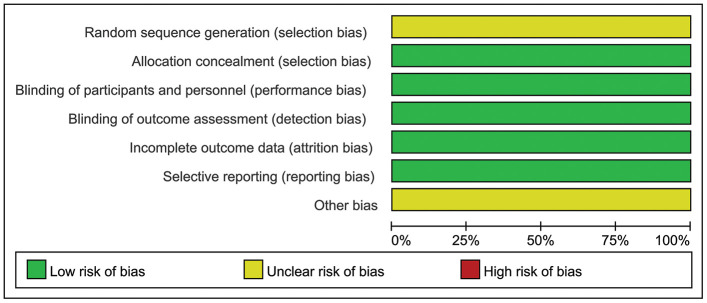
**Representation of potential bias in the included RCTs.**

The trials involved a total of 733 patients: 492 received single-agent PARPi (OlympiAD, olaparib; EMBRACA, talazoparib) and 241 received TPC. The mean sample size was 366.5, ranging between 302 and 431. Regarding study quality, two domains had unclear risk of bias and five domains had low risk of bias; therefore, both RCTs were considered to have unclear risk of bias (Figure 2).

Tables 1 and 2 show the main characteristics of the RCTs. The two studies were randomized, controlled, open-label, multicenter international phase III trials with participation from 16–18 countries. The mean trial duration was 28.5 months (range, 15–42 months). In both RCTs, about half of the patients had TNBC; <30% of the participants had received platinum-based chemotherapy before participating in the RCTs.

**Table 1:** Randomised controlled trials included in the systematic review and network meta-analysis.

**Study**	**Trials**	**Clinical trial cycle (months)**	**data cutoff**	**Trial phase**	**No. of center**	**BRCA mutation type**	**HR**	**Stage of breast cancer**	**previous chemotherapy regimens for metastatic disease**	**previous cytotoxic regimens for advanced breast cancer**
Robson et al.2017	OlympiAD	15	Dec.9, 2016	III	18	BRCA1/BRCA2/both	positive/negative	metastatic breast cancer	no more than two	unknown
Litton et al.2018	EMBRACA	42	Sep.15, 2017	III	16	BRCA1/BRCA2	positive/negative	metastatic or local advanced breast cancer	unknown	no more than three

**Table 2:** Characteristics of eligible studies.

**Trials**	**Sample sizes**		**Age range**		**Drug type**		**Dosage and frequency**		**Crossover from control group to experimental group**
	**Experiment**	**control**	**Experiment**	**control**	**Experiment**	**control**	**Experiment**	**control**	
OlympiAD	205	97	22–76	24–68	Olaparib	PTC	300 mg twice daily	standard therapy: eribulin mesylate administered intravenously at a dose of 1.4 mg on day 1 and day 8, repeated every 21 days; or vinorelbine administered intravenously at a dose of 30 mg on day 1 and day 8, repeated every 21 days.	not permitted
EMBRACA	287	144	27–84	24–88	Talazoparib	PTC	1 mg orally once daily continuously	standard-therapy: capecitabine, eribulin, gemcitabine, or vinorelbine in continuous 21-day cycles	not permitted

PTC: Standard-Therapy Group.

Here, we included two RCTs fulfilling all the eligibility criteria, and there were no head-to-head studies assessing the differences between single-agent PARPi. Due to the absence of a loop connecting three arms, the node-splitting method was not calculated for all outcomes.

### Efficacy outcomes

**Figure 3:**
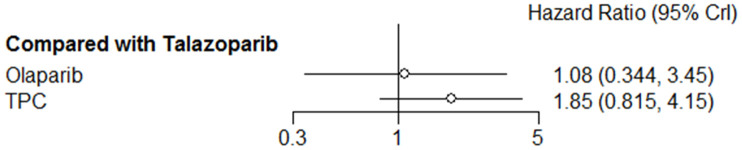
**Forest plot comparing PFS for talazoparib, olaparib, and TPC.**

Regarding efficacy, olaparib was not associated with improved PFS (*HR* = 1.08, 95% *CrI* = 0.34–3.45) when compared with talazoparib, and TPC conferred worse PFS (*HR* = 1.85, 95% *CrI* = 0.82–4.15). However, this was not statistically significant (Figure 3).

**Figure 4:**
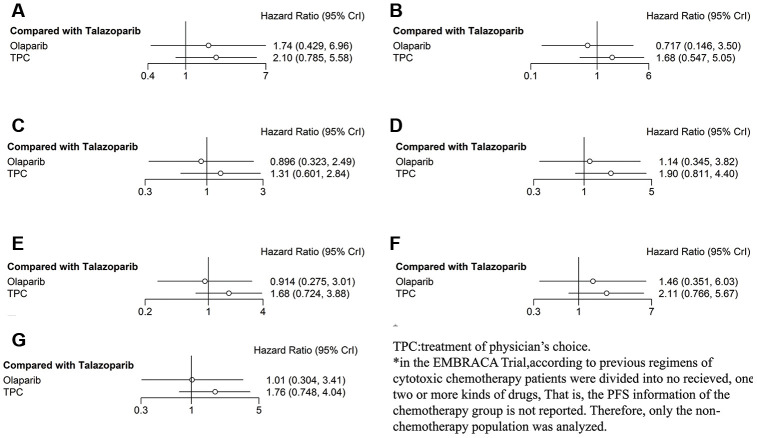
**Forest plots comparing PFS for talazoparib, olaparib, and TPC (subgroup analysis).** (**A**) Hormone receptor–positive patients; (**B**) patients with TNBC; (**C**) patients with prior platinum; (**D**) patients with no prior platinum; (**E**) patients with *BRCA1* mutation; (**F**) patients with *BRCA2* mutation; (**G**) patients who received no prior chemotherapy.

In subgroup analysis according to hormone receptor status, both olaparib and TPC conferred worse PFS in the hormone receptor–positive subgroup compared with talazoparib, but statistical significance was not obtained (olaparib: *HR* = 1.74, 95% *CrI* = 0.43–6.69; TPC: *HR* = 2.10, 95% *CrI* = 0.79–5.58) (Figure 4A). In analyses of patients with TNBC, olaparib was associated with improved PFS (*HR* = 0.72, 95% *CrI* = 0.15–3.50), while PFS was worst with TPC (*HR* = 1.68, 95% *CrI* = 0.55–5.05) (Figure 4B). In the subgroup analysis according to prior exposure to platinum-based chemotherapy, olaparib was not associated with PFS (olaparib: *HR* = 0.90, 95% *CrI* = 0.32–2.49) compared with talazoparib, and PFS was worst with TPC (*HR* = 1.31, 95% *CrI* = 0.60–2.84) (Figure 4C). When analyzing patients in the no prior platinum subgroup, PFS was worse with both olaparib and TPC (olaparib: *HR* = 1.14, 95% *CrI* = 0.35–3.82; TPC: *HR* = 1.90, 95% *CrI* = 0.81–4.40) (Figure 4D).

In the subgroup analysis according to *BRCA* mutation type, olaparib was not associated with PFS, and PFS was worse with TPC in the *BRCA1* mutation subgroup (olaparib: *HR* = 0.91, 95% *CrI* = 0.28–3.01; TPC: *HR* = 1.68, 95% *CrI* = 0.72–3.88) compared with talazoparib (Figure 4E), both results did not reach statistical significance. When exploring the difference between subjects with *BRCA1* mutation, PFS was worse with both olaparib and TPC (olaparib: *HR* = 1.46, 95% *CrI* = 0.35–6.03; TPC: *HR* = 2.11, 95% *CrI* = 0.77–5.67), although significant associations were not recognized (Figure 4F). Similar results were obtained when we analyzed only the subjects who had not received prior chemotherapy (olaparib: *HR* = 1.01, 95% *CrI* = 0.30–3.41; TPC: *HR* = 1.76, 95% *CrI* = 0.75–4.04) (Figure 4G).

**Figure 5:**
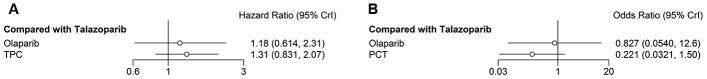
**Forest plots comparing OS and ORR for talazoparib, olaparib, and TPC. (A) OS; (B) ORR.**

We defined OS and ORR as secondary efficacy outcomes: when compared with talazoparib, the NMA of OS showed that PFS might be worse with olaparib and TPC, but the differences were short of statistical significance (olaparib: *HR* = 1.18, 95% *CrI* = 0.61–2.31; TPC: *HR* = 1.31, 95% *CrI* = 0.83–2.07) (Figure 5A). Regarding ORR, compared with talazoparib, both olaparib and TPC were associated with non-significantly improved ORR (olaparib: *OR* = 0.83, 95% *CrI* = 0.05–12.64; TPC: *OR* = 0.22, 95% *CrI* = 0.03-1.50) (Figure 5B).

### Safety

**Figure 6:**
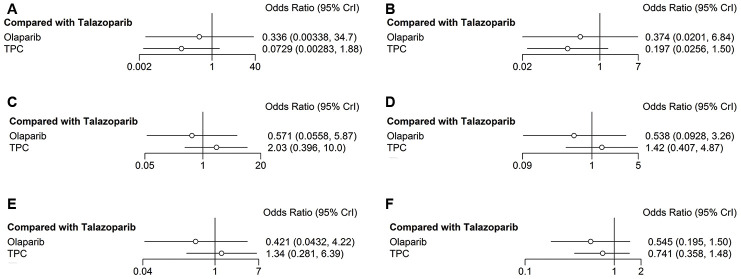
**Forest plots comparing any-grade and grade 3–4 hematological AEs.** (**A**) Grade 3–4 anemia; (**B**) any-grade anemia; (**C**) grade 3–4 neutropenia; (**D**) any-grade neutropenia; (**E**) grade 3–4 decreased white cell count; (**F**) any-grade decreased white cell count.

Here, major hematologic AEs were defined as primary safety outcome, and included anemia, neutropenia, and decreased white cell count. In the olaparib trial, grade 3–4 anemia was reported in 82 of 205 patients in the olaparib group and in 24 of 91 patients in the TPC group. In the talazoparib trial, grade 3–4 anemia was reported in 151 of 286 patients in the talazoparib group and in 23 of 126 patients in the control group. Compared with talazoparib, olaparib and TPC reduced the risk of developing grade 3–4 anemia (olaparib: *OR* = 0.34, 95% *CrI* = 0.003–34.94; TPC: *OR* = 0.07, 95% *CrI* = 0.003–1.86) (Figure 6A) and any-grade anemia (olaparib: *OR* = 0.37, 95% CrI = 0.02–6.81; TPC: *OR* = 0.20, 95% *CrI* = 0.03–1.52) (Figure 6B). Regarding neutropenia, olaparib showed grade 3–4 neutropenia compared with talazoparib (*OR* = 0.57, 95% *CrI* = 0.06–5.75), while TPC showed worse results (*OR* = 2.00, 95% *CrI* = 0.40–9.99) (Figure 6C). Similar results were obtained for any-grade neutropenia (olaparib: *OR* = 0.54, 95% *CrI* = 0.09–3.27; TPC: *OR* = 1.42, 95% *CrI* = 0.41–4.95) (Figure 6D). Similarly, olaparib had better decreased white cell count (grade 3–4) compared with talazoparib (*OR* = 0.42, 95% *CrI* = 0.04–4.19), while TPC had the worst result (*OR* = 1.32, 95% *CrI* = 0.28–6.19) (Figure 6E). The results were altered when we reanalyzed any-grade decreased white cell counts: Olaparib and TPC had better decreased white cell counts (olaparib: *OR* = 0.55, 95% *CrI* = 0.20–1.52; TPC: *OR* = 0.75, 95% *CrI* = 0.37–1.49) (Figure 6F).

**Figure 7:**
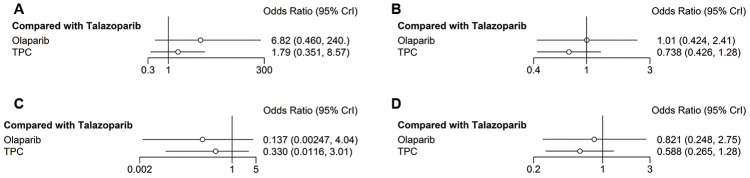
**Forest plots comparing any-grade and grade 3–4 non-hematological/any AEs.** (**A**) Grade 3–4 fatigue; (**B**) any-grade fatigue; (**C**) grade 3–4 headache; (**D**) any-grade headache.

The secondary safety outcome mainly included fatigue and headache. Compared with talazoparib, olaparib and TPC were associated with increased risk of grade 3–4 fatigue (olaparib: *OR* = 6.79, 95% *CrI* = 0.44-262.48; TPC: *OR* = 1.83, 95% *CrI* = 0.35–8.64) (Figure 7A). Regarding any-grade fatigue, olaparib and TPC did not show any significant difference compared with talazoparib (olaparib: *OR* = 1.01, 95% *CrI* = 0.42–2.40; TPC: *OR* = 0.74, 95% *CrI* = 0.43–1.28) (Figure 7B). Regarding headache, olaparib and TPC had better grade 3–4 headache compared with talazoparib (olaparib: *OR* = 0.14, 95% *CrI* = 0.003–4.17; TPC: *OR* = 0.35, 95% *CrI* = 0.01–3.18) (Figure 7C). Similar results were obtained for any-grade headache (olaparib: *OR* = 0.82, 95% *CrI* = 0.25–2.74; TPC: *OR* = 0.59, 95% *CrI* = 0.27–1.28) (Figure 7D).

### Acceptability

**Figure 8:**
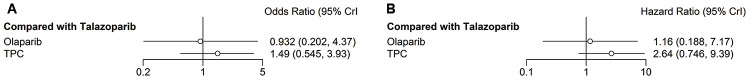
**Forest plots comparing acceptability of treatment discontinuation and QoL.** (**A**) Treatment discontinuation; (**B**) QoL.

Regarding acceptability, the primary and secondary outcome was treatment discontinuation rate and time to QoL deterioration, respectively. Treatment was discontinued due to any-grade AEs by 10 and 7 patients in the olaparib and TPC group, respectively, and by 17 and 11 patients in the talazoparib and TPC group, respectively. Similarly, compared with talazoparib, olaparib did not show any significant difference for treatment discontinuation (*OR* = 0.93, 95% *CrI* = 0.20–4.37), and TPC conferred higher risk, while no significant differences were found (*OR* = 1.49, 95% *CrI* = 0.55–3.93) (Figure 8A).

Regarding time to QoL deterioration, olaparib and TPC were associated with short time to clinically meaningful QoL deterioration compared with talazoparib (olaparib: *HR* = 1.16, 95% *CrI* = 0.19–7.17; TPC: HR = 2.64, 95% CrI = 0.75–9.39), while no significant difference was obtained (Figure 8B).

## DISCUSSION

We conducted an NMA to compare the efficacy, safety, and acceptability of single-agent PARPi. To the best of our knowledge, ours is the first NMA to compare the effect of different treatments with single PARPi in patients with advanced breast cancer. Both included articles showed that single-agent PARPi conferred a significant benefit over TPC for PFS. The NMA showed no statistically significant difference between olaparib and talazoparib in terms of efficacy, safety, and acceptability, which means that the two FDA-approved single-agent PARPi have similar efficacy, safety, and acceptability when used for treating patients with advanced breast cancer. Another point worth mentioning is that PARP enzymes are essential in DNA damage repair. With defective homologous recombination DNA repair, *BRCA1*- and *BRCA2*-mutated breast cancers are targets for PARPi through the exploitation of synthetic lethality. Therefore, PARPi confer an advantage in breast cancer therapy based on biological principles.

However, some limitations of the present NMA should be acknowledged. We included only two RCTs in our study. Further studies should attempt to expand the sample size for in-depth re-analysis by including more RCTs. Nevertheless, we deeply believe that the analyses performed may aid in presenting a brief assessment of the role of single-agent PARPi for managing patients with *BRCA*-mutated *HER2*-negative advanced breast cancer.

In conclusion, any single-agent PARPi has similar efficacy, safety, and acceptability in patients with *BRCA*-mutated *HER2*-negative metastatic or advanced breast cancer. Although the optimal choice of single-agent PARPi, as well as the possible combination strategies with other therapies, has not been determined, single-agent PARPi can now be regarded as a similar option in the therapeutic armamentarium for patients with *BRCA*-mutated *HER2*-negative metastatic or advanced breast cancer.

## MATERIAL AND METHODS

### Search strategy and selection criteria

This study is registered with PROSPERO (CRD42019138939) and is reported according to the Preferred Reporting Items for Systematic Reviews and Meta-Analyses (PRISMA) extension statement for NMA [16].

For this NMA, we searched Web of Science, Embase, PubMed, Medline, ClinicalTrials.gov, the Cochrane Central Register of Controlled Trials, and the World Health Organization (WHO) International Clinical Trials Registry Platform (ICTRP) from the date of inception to July 20, 2019, with no language restrictions. References derived from full text review were screened to identify potential randomized controlled trials (RCTs) that had not been indexed in the above databases.

We used a prespecified search strategy using terms applicable to the population of interest. The search strategy was designed using a combination of medical subject headings (MeSH) and keywords. The MeSH and keywords included “breast”, “ mammary”, “cancer”, “cancers”, “tumor”, “neoplasm”, “carcinoma”, “poly(ADP-ribose) polymerase inhibitors”, “poly(ADP-ribose) polymerases”, “PARPi”, PARPi names (olaparib, veliparib, niraparib, rucaparib, talazoparib), and synonymous words. The reference lists from relevant studies, meta-analyses and reviews were manually screened for potentially eligible publications.

The electronic database searches were supplemented with manual searches for published, unpublished, and ongoing RCTs in international trial registers, drug approval agency websites, and key scientific journals in the field [13], and important professional conferences such as that of the American Society of Clinical Oncology (ASCO).

### Study selection

Four investigators (JW, YZ, LY, LR) selected the studies independently. The titles and abstracts of identified publications were screened; potentially eligible articles were retrieved for full-text review. Disagreements were resolved by discussion with a fifth author (XQ).

Eligible studies were phase II or III RCTs for which efficacy and/or safety outcomes were available. Studies that compared any single-agent PARPi with standard mono-chemotherapy in patients with *BRCA*-mutated *HER2*-negative metastatic or advanced breast cancer were included. Eligible patients were at least 18 years of age and had *HER2*-negative breast cancer that was hormone receptor–positive or was triple negative. Furthermore, patients with confirmed deleterious g*BRCA* mutation and previous neoadjuvant or adjuvant treatment with platinum were included. Study subjects were limited to those with metastatic or local advanced breast cancer. We only included double-blind trials, and included placebo in the NMA, and we increased the methodological rigor of the study design by minimizing performance and ascertainment biases [17].

### Data extraction and quality assessment

Four investigators (JW, YZ, LY, LR) independently extracted and summarized the data from all included studies using a standardized data abstraction form and assessed the risk of bias. The standardized data extraction forms included the trial characteristics (e.g., publication year, first author, journal), subject characteristics (e.g., the number of patients, patient age, previous treatment), intervention details (e.g., PARPi type, dose, duration of treatment), and outcome measures (efficacy, safety, acceptability).

The quality of the included RCTs was assessed using the Cochrane risk of bias tool [18] on selection, attrition, performance, detection, and reporting bias. Each RCT was deemed to be at low, high, or unclear risk of bias based on its adequacy of random sequence generation; allocation concealment; participant and personnel blinding; outcome assessor blinding; and method of addressing incomplete data, selective reporting, and other potential sources of bias. Trials with a rating of high risk of bias in one or more domains were considered high risk, trials with low risk of bias in all domains were low risk, and trials with unclear risk of bias in one or more domains were unclear risk [19]. Four investigators (JW, YZ, LY, LR) were separately and independently assigned for each RCT. Any discrepancies were resolved by consensus or by a fifth investigator (XQ).

### Outcomes

The outcomes of interest were efficacy, safety, and acceptability. The primary efficacy outcome of interest was progression-free survival (PFS), the primary safety outcome of interest was major all-grade and high-grade (grade 3–4) treatment-related adverse events (AEs), and the primary acceptability outcome of interest was discontinued treatment due to AEs. Major AEs were defined as hematologic adverse events (we only focused on anemia, neutropenia, and decreased white cell count), assessed by concomitant medications and clinically relevant changes in laboratory values. The secondary efficacy outcomes included overall survival (OS) and objective response rate (ORR). The secondary safety outcome was defined as all-grade and high-grade (grade 3–4) non-hematologic AEs (fatigue, headache); the secondary acceptability outcome was impact on quality of life (QoL). Decreased QoL was defined as patients with clinically meaningful deterioration of QoL, as evaluated by the global health status/QoL scale on the EORTC QLQ-C30 (European Organization for Research and Treatment of Cancer QoL of cancer patients questionnaire).

### Statistical analysis

NMA was carried out using a random-effect model within a Bayesian framework and executed by R software using the gemtc package (version 3.5.1; R Foundation, Vienna, Austria) [20, 21], which recalls JAGS in R for Markov chain Monte Carlo (MCMC) sampling. MCMC methods were applied to calculate the hazard ratios (HRs) or odds ratios (ORs) with 95% credible intervals (CrIs). For each analysis, the function mtc.run was used to generate samples through MCMC sampler. Initially, 10,000 simulations were set up for each chain as the burn-in period, yielding 100,000 iterations to obtain the HRs or ORs of model parameters, while four Markov chains were run simultaneously. The Brooks-Gelman-Rubin statistic and trace plots were assessed to check whether the model convergence was satisfactory. The number of iterations would not increase if the model convergence was sufficient [22]. If closed loops existed in the network of comparisons in the present study, estimating incoherence and network-plot were carried out [23]. Furthermore, the inconsistency of the model was calculated using the node-splitting method, which separates evidence on a particular comparison into direct and indirect evidence [24, 25]. Inconsistency between indirect and direct sources of evidence was statistically assessed globally and locally [26]. The mtc.anohe command of the gemtc package was applied to evaluate global heterogeneity via the heterogeneity variance parameter *I*^2^. If at least 10 legal studies were available, the Egger test and funnel plot were used to detect publication bias [27].

The pooled estimates of *HR*s with 95% *CrI*s were calculated for PFS, OS, and the time to QoL deterioration. *HR* < 1 indicated lower probability of developing PFS and OS events, and delayed time to QoL deterioration. The pooled estimates of *OR* with 95% *CrI*s were calculated for the effect for ORR, AEs, and treatment discontinuation. *OR* > 1 indicated lower ORR, AEs, and treatment discontinuation rates.

To determine whether the results were affected by study characteristics, we performed subgroup analyses for primary efficacy outcomes according to the following variables: previous chemotherapy, previous platinum-based therapy, hormone receptor status, and BRCA mutation type. Two-sided P-values of <0.05 were considered statistically significant.

### Ethical statement

All authors are accountable for all aspects of the work in ensuring that questions related to the accuracy or integrity of any part of the work are appropriately investigated and resolved.
